# Neutralisation of HIV-1 cell-cell spread by human and llama antibodies

**DOI:** 10.1186/s12977-014-0083-y

**Published:** 2014-10-02

**Authors:** Laura E McCoy, Elisabetta Groppelli, Christophe Blanchetot, Hans de Haard, Theo Verrips, Lucy Rutten, Robin A Weiss, Clare Jolly

**Affiliations:** Division of Infection and Immunity, University College London, London, WC1E 6BT United Kingdom; ArGEN-X BVBA, Zwijnaarde, Ghent Belgium; QVQ bv, 3584CH Utrecht, The Netherlands; Current address: Department of Immunology and Microbial Science, The Scripps Research Institute, La Jolla, CA 92037 USA

**Keywords:** HIV-1, Antibody, Virological synapse, Cell-cell, Neutralisation, CD4, VHH

## Abstract

**Background:**

Direct cell-cell spread of HIV-1 is a very efficient mode of viral dissemination, with increasing evidence suggesting that it may pose a considerable challenge to controlling viral replication *in vivo*. Much current vaccine research involves the study of broadly neutralising antibodies (bNabs) that arise during natural infection with the aims of eliciting such antibodies by vaccination or incorporating them into novel therapeutics. However, whether cell-cell spread of HIV-1 can be effectively targeted by bNabs remains unclear, and there is much interest in identifying antibodies capable of efficiently neutralising virus transmitted by cell-cell contact.

**Results:**

In this study we have tested a panel of bNAbs for inhibition of cell-cell spread, including some not previously evaluated for inhibition of this mode of HIV-1 transmission. We found that three CD4 binding site antibodies, one from an immunised llama (J3) and two isolated from HIV-1-positive patients (VRC01 and HJ16) neutralised cell-cell spread between T cells, while antibodies specific for glycan moieties (2G12, PG9, PG16) and the MPER (2F5) displayed variable efficacy. Notably, while J3 displayed a high level of potency during cell-cell spread we found that the small size of the llama heavy chain-only variable region (VHH) J3 is not required for efficient neutralisation since recombinant J3 containing a full-length human heavy chain Fc domain was significantly more potent. J3 and J3-Fc also neutralised cell-cell spread of HIV-1 from primary macrophages to CD4+ T cells.

**Conclusions:**

In conclusion, while bNabs display variable efficacy at preventing cell-cell spread of HIV-1, we find that some CD4 binding site antibodies can inhibit this mode of HIV-1 dissemination and identify the recently described llama antibody J3 as a particularly potent inhibitor. Effective neutralisation of cell-cell spread between physiologically relevant cell types by J3 and J3-Fc supports the development of VHH J3 nanobodies for therapeutic or prophylactic applications.

## Background

All currently licensed vaccines against human viruses induce humoral immunity and elicit antibodies that neutralise the virus, thereby preventing infection of host cells [1,2]. To date antibodies which broadly neutralise the many diverse strains of Human Immunodeficiency Virus Type-1 (HIV-1) have only been elicited during natural infection or in the heavy-chain only antibody (HCAb) format found in llamas [3]. Consequently, much HIV-1 vaccine research involves the study of broadly neutralising antibodies (bNabs) that arise in certain patients with the goal of re-eliciting such antibodies by vaccination. This work has primarily focused on the ability of such antibodies to prevent infection of target cells by cell-free virions, notably in the standardised TZM-bl assay [4], which has been instrumental in allowing comparison of antibodies isolated from different patients and at various stages of disease. However, infection of susceptible target cells is also achieved via cell-cell contact. The predominant mode of cell-cell spread of HIV-1 is across virus-induced immune cell contacts termed virological synapses [5] that constitute >90% of cell-cell transmission events *in vitro* [6], although longer range cell-cell transmission via filopodia [7] and membrane nanotubes have also been reported [8]. Notably, cell-cell spread of HIV-1 is significantly more efficient than equivalent cell-free infection [9-14], with the increased infection kinetics of cell-cell spread attributed to a combination of factors including polarised budding of the virus towards the target cell, receptor clustering on the target cell enriching for viral entry receptors, and the close physical contact between cells limiting the requirement for prolonged virus diffusion [5,10,14,15].

While the relative importance of these two mechanisms of infection is difficult to definitively determine, there is growing awareness that assessing only cell-free virus does not adequately reflect the viral challenge present during *in vivo* infection, particularly since lymphoid tissues which are densely-packed with CD4+ T lymphocytes and thus provide an ideal environment for efficient viral dissemination mediated by physical intercellular contacts. In addition to increasing infection kinetics, it has been argued that the higher concentration of virus that can be passed from an infected cell to an uninfected target cell is of such a magnitude that some anti-retroviral agents are not fully efficient at controlling *in vivo* infection despite strong *in vitro* potency [16,17]. Furthermore cell-cell spread of HIV-1 has also been suggested to be a means by which HIV-1 may evade neutralising antibodies, and it has been reported that antibodies targeting the CD4 binding site are less able to neutralise infection by cell-cell spread than antibodies targeting other sites on HIV-1 [18].

Multiple sites on the HIV-1 envelope protein (Env) are targeted by bNabs, however many antibodies target the conserved CD4 binding site on Env which the virus uses to bind CD4 and infect host cells (e.g. HJ16, VRC01, NIH45-46, PGV04, b12, J3) [3]. Thus, the CD4 binding site is a target of many vaccine strategies that aim to induce bNabs at a protective level in the vaccinee at the time of exposure [19]. That anti-CD4 binding site antibodies can be protective has been demonstrated by the passive transfer of b12 to non-human primates and resistance to subsequent viral challenge [20,21]. However, there are differences in the ability of anti-CD4 binding site antibodies to neutralise HIV-1 both in terms of breadth and potency, reflecting their maturation in different hosts in response to diverse stimuli and specific isolation methods. Recent advances in isolating and eliciting of bNAbs against HIV-1 has led to the identification of a number of new broad and potent antibodies targeting the CD4 binding site including VRC01, HJ16 and J3 [22-24]. J3 is particularly interesting because unlike other broad and potent antibodies that were isolated from HIV-1 infected individuals, J3 is a HCAb variable region (VHH) that was isolated from a llama immunised with recombinant gp140 from subtypes A and B/C [22]. Llamas and other camelids contain HCAbs of approximately 82 KDa in addition to conventional antibodies of approximately 145 KDa [25]. In the HCAb all antigen-binding function is encoded in the VHH, and as these small domains are both highly stable and soluble these mini-antibodies have potential as microbicides [26] and as molecular tools [27]. In addition, they allow us to examine the relative importance of antibody size for effective neutralisation during cell-cell spread by reconstituting the full-length HCAb parent antibody of J3.

In this study we have directly compared the relative efficacy of antibodies targeting different epitopes within HIV-1 Env for their ability to block cell-cell spread of HIV-1 between CD4+ T lymphocytes using a panel of antibodies including some not previously tested for inhibition of cell-cell spread (J3, HJ16 and PG9). We report that broad and potent neutralising anti-CD4 binding site antibodies can neutralise cell-cell transmission of HIV-1 while antibodies 2F5, 4E10, 2G12 and PG9/16 which target the membrane proximal region (MPER), a high mannose patch and the V1/V2 loop respectively [28-30] display variable efficacy. In particular we found that J3 potently blocked cell-cell spread between physiologically relevant cell types including HIV-1 infected and uninfected T cells as well as transmission from macrophages to T cells. Notably the full-length heavy chain reconstituted VHH (J3-Fc) more effectively neutralises HIV-1 infection mediated either by cell-free or cell-cell spread, demonstrating that its potency is not solely a function of the small size of the antigen-binding VHH.

## Results

### T cell-T cell spread of HIV-1 is sensitive to antibody-mediated inhibition

**Figure 1:**
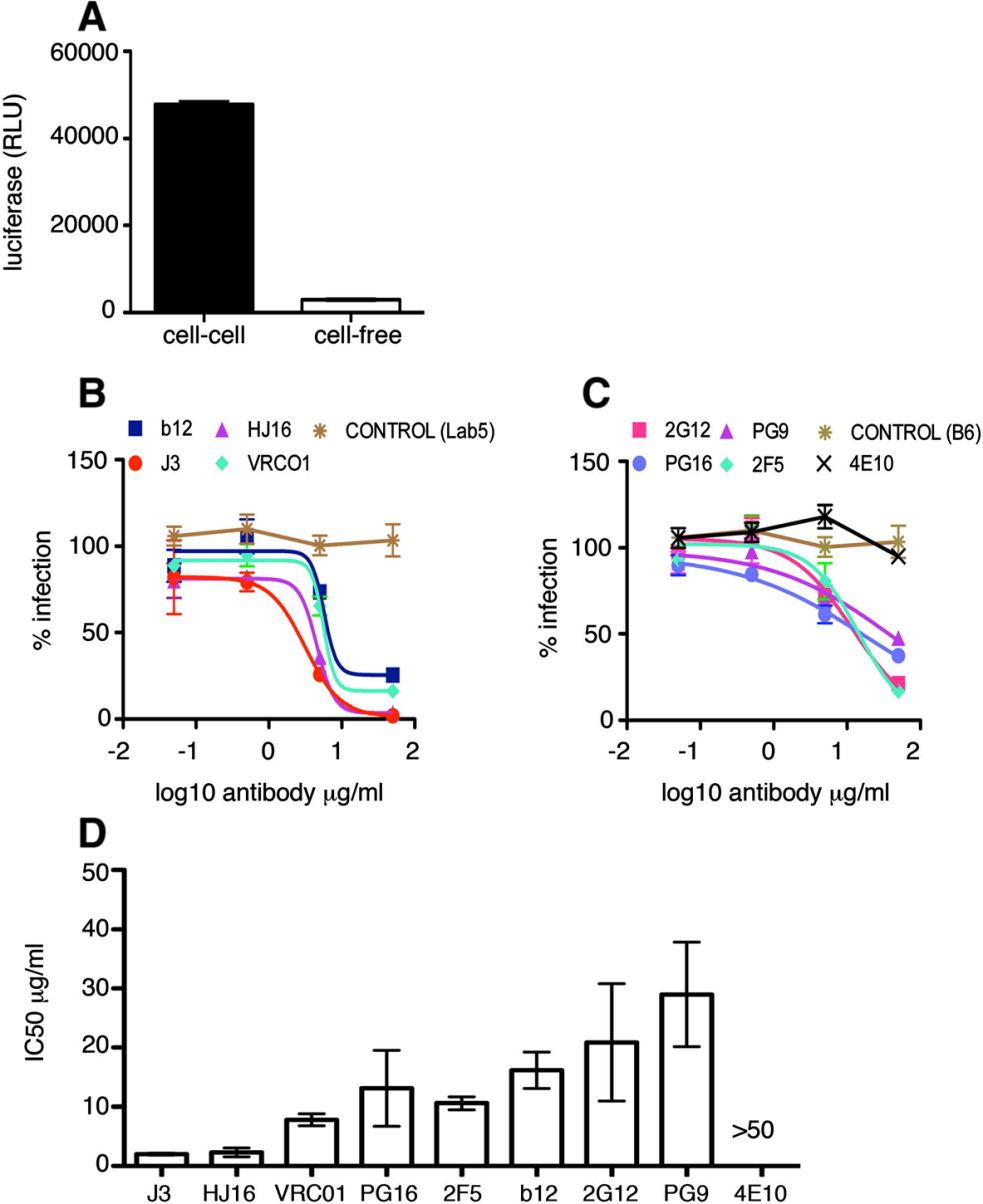
**Inhibition of HIV-1 T cell-T cell spread by anti-CD4 binding site, MPER and glycan-specific antibodies. (A)** Quantification of the luciferase signal in Jurkat 1G5 cells contributed by cell-cell and cell-free spread as described in the methods. Data show the mean and SEM from 3 independent experiments. **(B)** Antibodies targeting the CD4 binding site, **(C)** the gp41 MPER and gp120 glycans and non neutralising antibody controls (B6 and Lab5), were serially diluted and incubated with HIV-1 (NL4.3) infected Jurkat T cells for 1 h at 37°C. Uninfected target T cells containing a luciferase-reporter gene were added and cells incubated for 24 h as described in the methods to allow for cell-cell spread of HIV-1. Data are shown as the percentage neutralisation normalised to virus-only controls and representative of three independent experiments. **(D)** The average IC50 values (μg/ml) were generated from duplicate titrations of the indicated antibodies in three independent experiments and show the mean with the SEM.

We compared a group of bNabs targeting different epitopes on HIV-1 Env for their ability to inhibit cell-cell spread of HIV-1 between T cells. Notably, we evaluated inhibition of cell-cell spread by the recently described J3 VHH. J3 is a potent and broad inhibitor of cell-free HIV-1 infection [22] that is currently being evaluated as a potential microbicide in macaque challenge studies; however, whether J3 displays similar potency during cell-cell spread of HIV-1 has not been tested. To directly compare different antibodies, Jurkat T cells were infected with HIV-1 by spinoculation to achieve a synchronised population of infected cells 48 h post infection. For inhibition assays, infected Jurkat cells were incubated with serial dilutions of each antibody for 1 h at 37°C, and then mixed with uninfected target Jurkat cells that contain an HIV-1 tat-inducible luciferase gene (Jurkat 1G5). Luciferase reporter gene expression was measured after 24 h, thereby allowing direct quantification of new HIV-1 infection. This is a time frame in which the faster infection kinetics of cell-cell spread means this mode of viral spread dominates, with little or no contribution from cell-free virus [9,10,13,14,31] (Figure 1A) or syncytia formation [32]. Figure 1B shows that all four anti-CD4 binding site bNabs tested (b12, VRC01, HJ16 and J3) neutralised infection of T cells by cell-cell spread when compared to a non-neutralising human antibody control (B6 [33]) or the VHH negative control Lab5 [34]. Determination of the 50% inhibitory concentration (IC50) (Figure 1D) revealed that J3 was the most potent inhibitor of cell-cell spread (IC50 = 2 μg/ml) followed by HJ16 (IC50 = 2.3 μg/ml) and VRC01 (IC50 = 7.8 μg/ml). Notably, b12 was less potent compared to the other antibodies against this mode of transmission (IC50 = 16.1 μg/ml).

In addition three glycan-dependent and two anti-gp41 MPER antibodies were also assessed for inhibition of cell-cell spread across the same concentration range (Figure 1C and 1D). 2G12 was the first anti-glycan HIV-1 neutralising antibody described [35,36] and binds a high mannose cluster on gp120 through an unusual domain-exchanged structure [37]. Figure 1C shows that 2G12 also inhibited cell-cell spread of HIV-1, albeit with a higher IC50 than CD4 binding site bNabs J3, HJ16 and VRC01 (Figure 1D). PG9 and PG16 neutralise up to 80% of strains tested [28] via a glycan-dependent V1/V2 epitope [38] and we found that these bNabs also prevented cell-cell transmission but required a higher concentration (IC50 = 29 μg/ml and 13.1 μg/ml for PG9 and PG16 respectively) to achieve equivalent inhibition when compared to J3 or HJ16, with PG16 exhibiting greater potency than PG9 (Figure 1C and D). Finally two MPER-specific HIV-1 neutralising antibodies were also tested. Of these, only 2F5 was able to prevent cell-cell infection. 4E10 which has been reported to be less effective at blocking cell-free infection when compared to 2F5 [39], showed no inhibition of cell-cell spread (Figure 1C and D) when used at the highest concentration tested, giving an IC50 in excess of >50 μg/ml.

**Figure 2:**
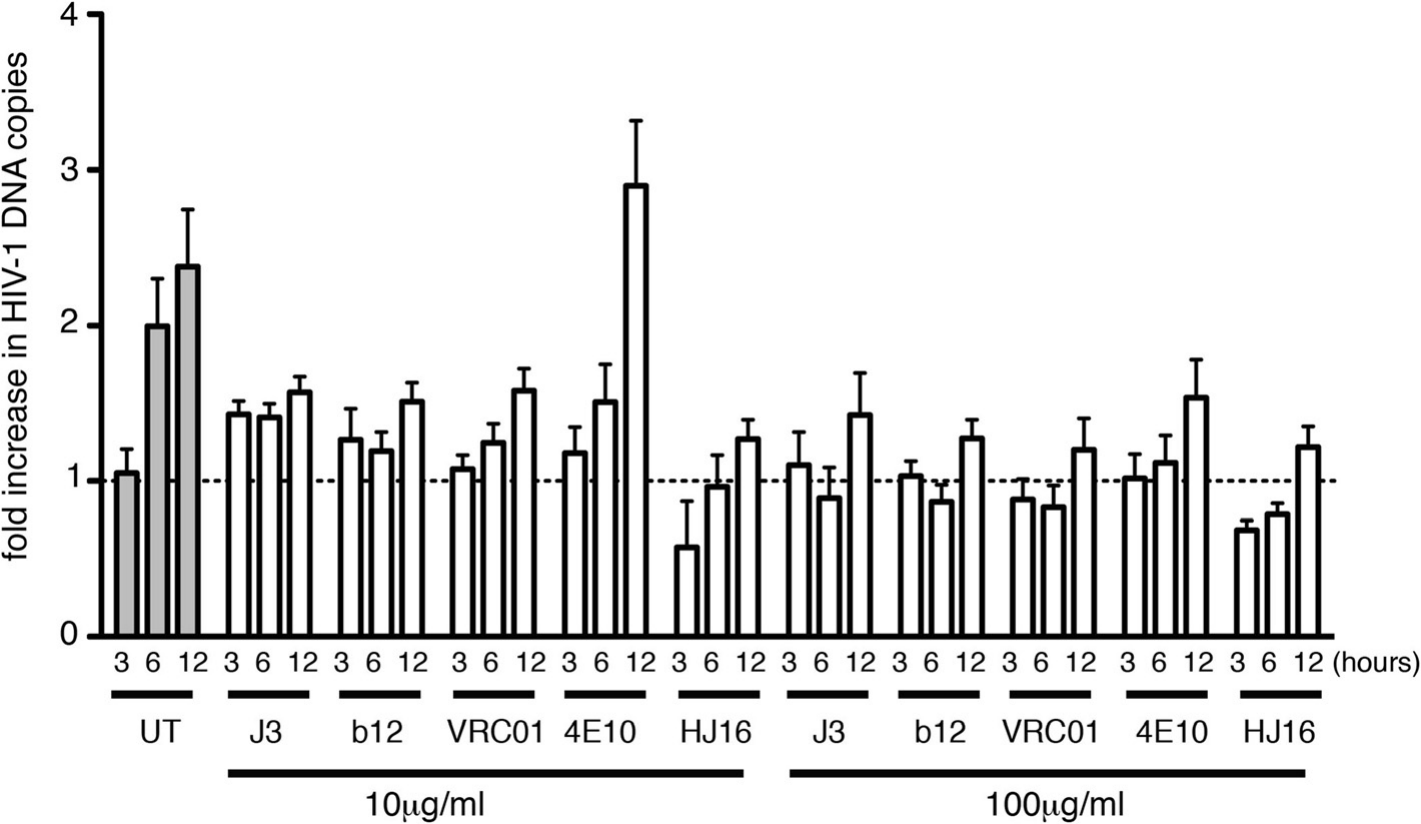
**Inhibition of cell-cell spread assayed by quantitative real-time PCR.** HIV-1 (NL4.3) infected Jurkat cells were either left untreated (control) or incubated with 10 or 100 μg/ml of the indicated antibodies for 1 h at 37°C prior to mixing with uninfected Jurkat 1G5 target T cells. DNA was extracted at 0, 3, 6 and 12 h and quantitative real-time PCR was performed to measure the appearance HIV-1 *pol* DNA resulting from *de novo* reverse transcription in newly-infected target cells. Data were normalised to the *albumin* housekeeping gene and expressed as the fold increase in HIV-1 DNA copy number over time relative to the baseline value at t = 0 h. Value of greater than 1 indicates an increase in HIV-1 DNA. Data are the mean with SD from a representative of 2 independent experiments. Statistical analysis was performed using by Anova with Tukey’s post-test to compare each antibody to the control (untreated) at corresponding time points (t = 3 h, t = 6 h and t = 12 h). Values for J3, b12 VRC01, and HJ16 were significantly different from untreated controls at 6 h and 12 h at both antibody concentrations (p < 0.001). 4E10 was significantly different from untreated controls at 6 h and 12 h only when used at 100 μg/ml (p < 0.001).

We next used a different assay system that employs quantitative real-time PCR (qPCR) to measure cell-cell spread by enumerating the appearance of *de novo* HIV-1 DNA *pol* copies arising from reverse transcription within the newly infected T cell population. The details and validation of this assay have been described elsewhere [9,10]. HIV-1 infected T cells were pre-incubated with bNabs at 10 μg/ml or 100 μg/ml for 1 h at 37°C, mixed with uninfected target cells and incubated for various periods of time, after which the DNA was extracted and qPCR performed. As expected, we observed a time-dependent increase in the appearance of HIV-1 *pol* DNA indicative of cell-cell spread within the control sample (UT) that was incubated in the absence of antibody (Figure 2). Notably, no statistically significant increase in the HIV-1 *pol* copy number was seen in the presence of the CD4 binding site bNabs J3, b12, VRC01 or HJ16 when used at either 10 μg/ml or 100 μg/ml, indicative of impaired cell-cell spread of HIV-1 in the presence of these antibodies (*p* <0.001). 4E10 again showed inefficient inhibition of cell-cell spread at 10 μg/ml when compared to other antibodies tested, and gained statistically significant inhibitory activity only when used at a 10-fold higher concentration.

Commonly used assays to evaluate neutralising activity of antibodies raised against HIV-1 are performed by titrating cell-free virus on HeLa TZM-bl reporter cells. Therefore, we compared the relative inhibitory activity of our panel of CD4-binding site antibodies during cell-free infection using the standardised TZM-bl assay. Table 1 shows that all antibodies targeting the CD4 binding site (J3, b12, VRC01 and HJ16) inhibited cell-free virus infection with IC50 values below 1 μg/ml, with J3 exhibiting approximately 10-fold higher potency than b12. This is similar to cell-cell spread (Figure 1) in which b12 was found to be 2–8 fold less effective than J3 and also other antibodies targeting the CD4 binding site (i.e. VRC01 and HJ16). Antibodies targeting other epitopes of Env (MPER, V1/V2 and glycans) generally showed reduced efficacy. Taken together these data show J3 to be an effective inhibitor of both modes of HIV-1 dissemination.

**Table 1:** **IC50 values** ^**a**^ **for inhibition of cell-free infection by bNabs**

	**J3**	**HJ16**	**VRC01**	**b12**	**2F5**	**4E10**	**2G12**	**PG9**	**PG16**
**Cell-free**	0.035	0.25	0.26	0.32	1.2	3.1	2.2	2.18	0.45
**SEM**	0.005	0.02	0.09	0.035	0.5	1.0	0.8	1.02	0.36

^a^IC50 values are μg/ml and were determined from at least three independent experiments as described in the Methods.

### Neutralisation of cell-cell spread mediated by HIV-1-infected primary T cells

**Figure 3:**
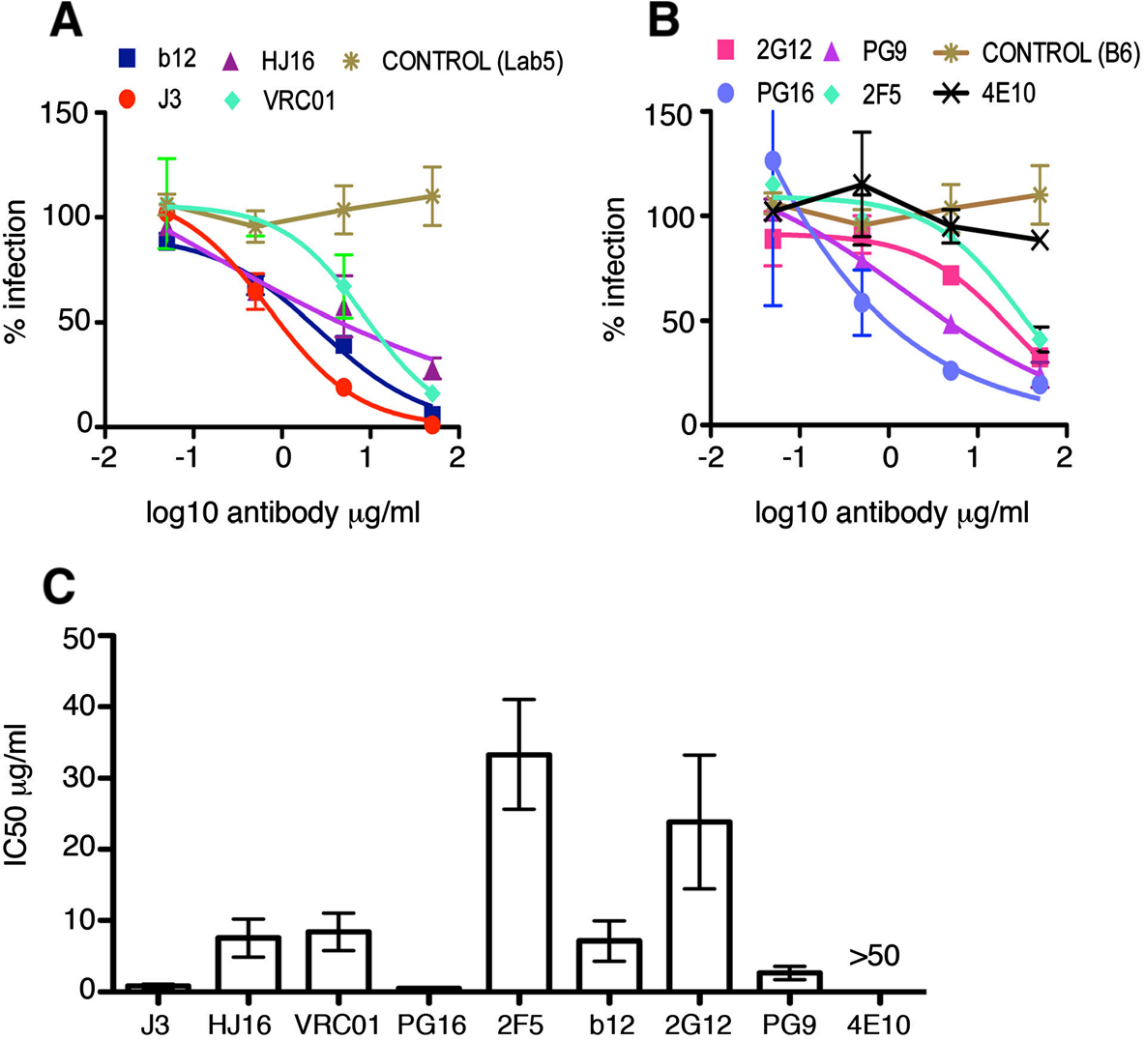
**Neutralisation of cell-cell spread by HIV-1 infected primary CD4+ T cells.** Antibodies targeting the CD4 binding site **(A)**, the MPER and gp120 glycans **(B)**, and non neutralising antibody controls (B6 and Lab5), were serially diluted and incubated with HIV-1 (NL4.3) infected primary CD4+ T cells for 1 h at 37°C exactly as in Figure 1. Uninfected target T cells containing a luciferase-reporter gene were added and cells incubated for 24 h as described in the methods to allow for cell-cell spread of HIV-1. Data are shown as the percentage neutralisation normalised to virus-only controls and representative of three independent experiments. **(C)** The average IC50 values (μg/ml) were generated from duplicate titrations of the indicated antibodies in three independent experiments and show the mean with the SEM.

While Jurkat T cells provide a reliable model in which to evaluate inhibition, we wished to confirm our data using primary CD4+ T cells, which are the main cell type infected by HIV-1 during natural infection. To this end CD4+ T cells were isolated from peripheral blood mononuclear cells, stimulated with PHA and IL-2 and infected with HIV-1 by spinoculation. After 48 h, HIV-1 infected primary CD4+ T cells were incubated with serial dilutions of antibodies and mixed with Jurkat 1G5 target cells and cell-cell spread quantified by luciferase assay exactly as described in Figure 1. Similar to the results of the Jurkat-Jurkat cell assay, all four anti-CD4 binding site bNabs were able to block HIV-1 cell-cell spread from primary HIV-1 infected T cells to target Jurkat 1G5 T cells (Figure 3A). Notably, we again found that J3 was the most potent neutralising antibody tested (IC50 = 0.80 μg/ml), with HJ16 and VRC01 inhibiting with an IC50 values of 7.55 μg/ml and 8.42 μg/ml respectively (Figure 3C). In this assay, b12 appeared to be slightly more potent against primary CD4+ T cell mediated infection (Figure 3A and C) compared to Jurkat cells (IC50 = 7.13 μg/ml in primary cells and 16.14 μg/ml in Jurkat cells) but like other CD4-binding site antibodies it also showed reduced potency compared to J3. Interestingly, the V1/V2 glycan-specific antibodies PG9 and PG16 showed increased activity against the primary CD4+ T cell associated virus during cell-cell infection (IC50 = 0.60 μg/ml and 0.26 μg/ml). 2G12 on the other hand showed a broadly consistent level of neutralisation whether the HIV-1 infected cells were primary or immortalised T cells (Figure 3B). The MPER-specific bNAb 2F5 was relatively less active against primary T cell-associated virus, and again 4E10 was ineffective at blocking cell-cell spread (Figure 3B).

### Efficient neutralisation by J3 is not dependent on antibody size

**Figure 4:**
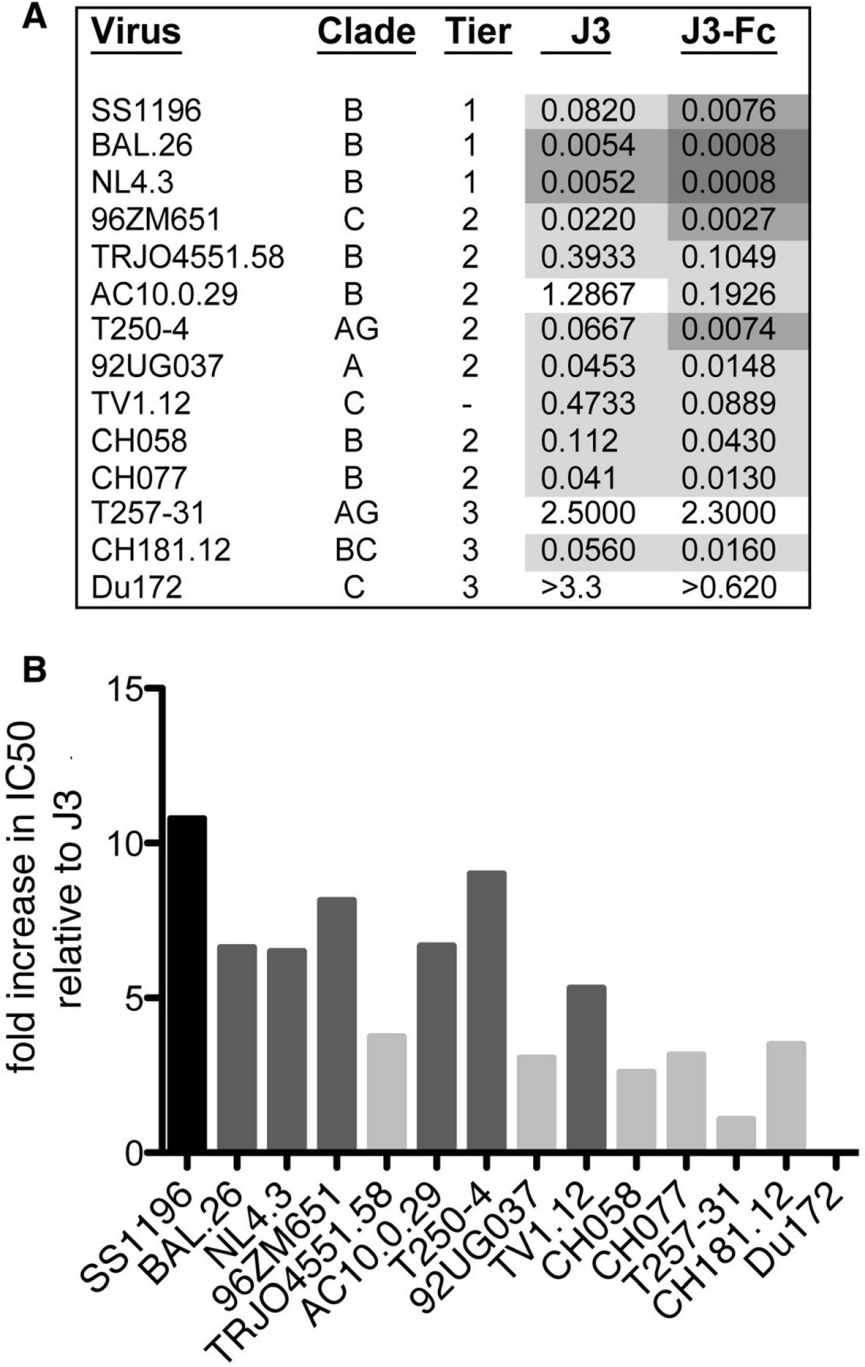
**Enhanced potency of full-length HCAb antibodies. (A)** IC50μM for the mono-specific bivalent J3-Fc in comparison to J3 alone for the viruses listed. Very potent neutralisation (<0.001) is indicated in dark grey; potent neutralisation (0.001-0.01) in grey, intermediate neutralisation in light grey (0.01-0.5) and weak neutralisation in white (>0.5). IC50 values were generated from duplicate titrations of J3 and J3-Fc onto TZM-bl cells as described in the methods. **(B)** Fold increase in IC50 for J3-Fc relative to J3 for indicated viruses. The subtype/CRF of each individual virus is indicated on the X axis.

It is plausible that the small size of the VHH J3 could be responsible, at least in part, for the high potency with which J3 blocked HIV-1 infection relative to the other CD4-binding site antibodies we tested. However, when the IC50 values for inhibition of cell-cell spread mediated by primary CD4+ T cells were compared on a molar scale we found that J3 remained the most potent of the anti-CD4 binding site antibodies tested (IC50 J3 = 0.055 μM +/−0.018; HJ16 = 0.104 μM +/− 0.036; VRC01 = 0.116 μM +/−0.036; b12 = 0.098 μM +/−0.038), with only PG9 and PG16 showing similar or better activity (PG9 = 0.037 μM +/−0.013; PG16 = 0.0069 μM +/−0.002). To directly consider the impact of size on neutralisation by J3, J3 VHH DNA was cloned upstream of a human immunoglobulin (IgG) CH2-CH3 region (J3-Fc), expressed in HEK 293 cells and purified by affinity to protein A. This process allowed reconstruction of J3 into the original heavy chain only format in which it was elicited in response to immunisation [22] but with a human Fc region. First we assessed cell-free neutralisation by J3 and J3-Fc against a panel of viruses that were either sensitive or resistant to neutralisation by the VHH J3 monomer in the TZM-bl assay (Figure 4A). In all cases where monomeric VHH J3 neutralised a viral strain, the IC50 (μM) of the VHH-Fc was more potent than that for the individual VHH. However, where the monomeric J3 was unable to neutralise a particular strain, the corresponding J3-Fc was also unable to neutralise. For example, Du172.17 is resistant to both J3 and J3-Fc. Notably, for all but one strain, the J3-Fc was more potent than the J3 monomer resulting in a decrease in IC50 of between two- and eleven-fold for thirteen out of fourteen viruses tested (Figure 4B). That these hybrid full-length HcAb neutralise a variety of HIV-1 strains across a range of clades and tiers, generally with enhanced potency, strongly suggests that the immunisation-elicited parental HcAbs from which J3 was derived would have similar potent neutralisation ability, and negates the hypothesis that these VHH neutralise HIV effectively due to their smaller size.

**Figure 5:**
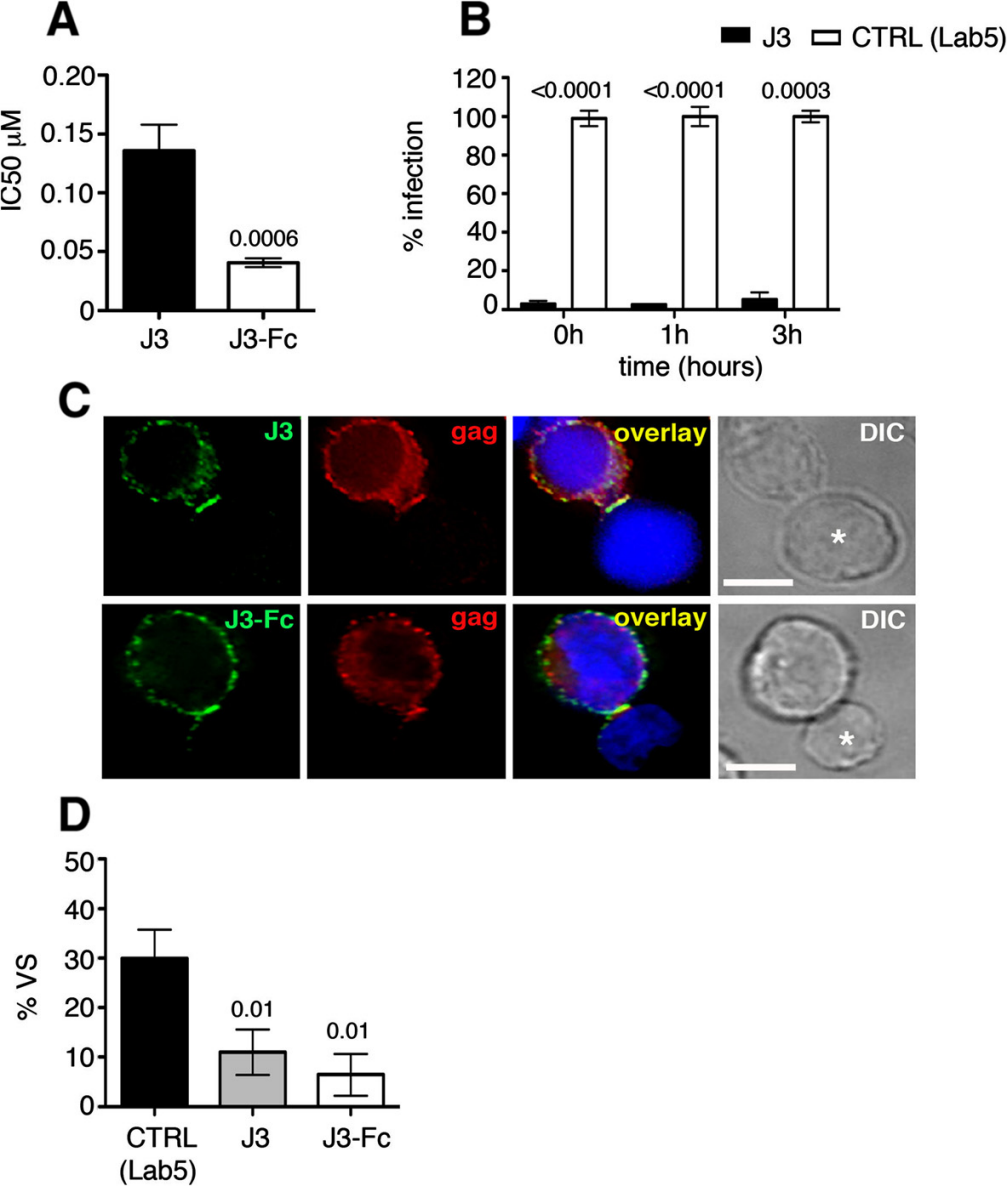
**Antibody size is not a limit to neutralisation of cell-cell spread by J3. (A)** IC50 values (μM) generated from duplicate titrations of the J3 VHH and full length heavy chain only J3 (J3-Fc) incubated for 1 h with HIV-1 (NL4.3) infected Jurkat cells and then mixed with target 1G5 luciferase-encoding cells for 24 h as for Figure 1. Student’s *t*-test was performed using data from 3 independent experiments. **(B)** The percentage cell-cell spread when J3 was added during (t = 0 h) or after (t = 1 h and t = 3 h) mixing HIV-1 infected Jurkat cells and uninfected 1G5 target cells. Statistical analysis was performed using a student’s *t*- test. **(C)** Immunofluorescence staining showing J3 (top panel) and J3-Fc (bottom panel) can access the virological synapse formed during cell-cell contact between a Gag + HIV-1 infected T cell and an uninfected target T cell (asterisk). Gag (red), J3 (green) and DAPI (blue). Scale bar is 5 microns. **(D)** Inhibition of virological synapse formation by J3 and J3-Fc. HIV-1 (NL4.3) infected T cells were mixed with uninfected T cells in the presence of J3, J3-Fc or control (Lab5) and incubated for 1 h at 37°C. Cells were then fixed and permeabilised, stained for HIV-1 Gag and imaged by immunofluorescence microscopy. Random fields were selected and the number of synapses formed between HIV-1 infected T cells and uninfected target T cells (defined by polarisation of Gag to the cell-cell interface) were scored. Data are the mean and SEM from 3 independent experiments. Statistical analysis was performed by comparing to control Lab5 using an Anova with Tukey’s post-test.

Next we compared J3 VHH (14.5 kDa) and J3-Fc (82 kDa) for inhibition of cell-cell spread. Figure 5A shows that both J3 and J3-Fc potently neutralised the cell-cell spread of HIV-1 with IC50 values of 0.136 μM and 0.040 μM respectively. The increased potency of J3-Fc, when considered on a molar scale, is highly statistically significant (*p* = 0.0006) and is consistent with data obtained when evaluating J3-Fc neutralisation of cell-free infection against multiple viruses (Figure 4). Thus we conclude that efficient neutralisation of cell-cell spread by J3 when compared to other antibodies is not simply due to its smaller size.

### J3 inhibits virological synapse formation

The predominant mode of HIV-1 cell-cell spread is across virus-induced virological synapses [5,40] that form at sites of physical cell-cell contact. It has previously been shown that virological synapses are dynamic contacts with a mean contact time of 62 min and that antibody targeting Env-CD4 binding can prevent HIV-1 infection when added up to 3 h after mixing infected and uninfected cells [10]. To determine whether J3 can also neutralise cell-cell spread after synapse formation, we added the antibody at the time of mixing infected and uninfected cells (t = 0 h) or 1 h and 3 h post-mixing. Strikingly, the percentage neutralisation mediated by 10 μg/ml of J3 was maintained whether the antibody was added during (t = 0) or after mixing (t = 1 h and t = 3 h) (Figure 5B), similar to previous reports with human IgG [10].

The HIV-1 T cell virological synapse is defined by enrichment of HIV-1 Gag and Env at sites of cell-cell contact and is dependent on receptor-mediated intercellular interactions. Indeed, studies have shown that antibodies against HIV-1 Env that target the CD4 binding site can access the cell-cell interface and inhibit synapse formation [5,9,12]. Therefore, having shown that both J3 and J3-Fc can block cell-cell spread we next investigated whether these antibodies inhibit synapse formation. Immunofluorescence microscopy staining revealed that both J3 and J3-Fc could be detected at intercellular junctions formed between HIV-1 infected and uninfected T cells, engage HIV-1 Env and stain the cell-cell interface (Figure 5C). Quantification of synapse formation revealed that both J3 and J3-Fc significantly decreased the percentage of virological synapses that formed between HIV-1 infected and uninfected T cells by 65% and 85% respectively (Figure 5D).

### Neutralisation of cell-cell spread between primary T cells

**Figure 6:**
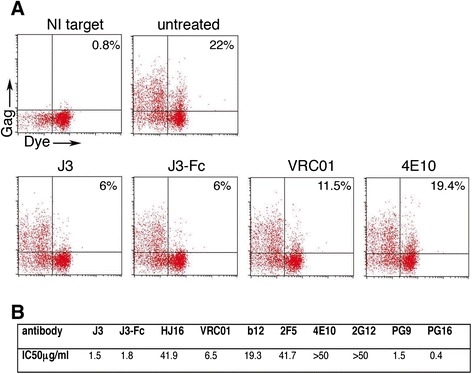
**Neutralisation of cell-cell spread between autologous primary T cells.** Antibodies targeting the CD4 binding site, the MPER and gp120 glycans were serially diluted and incubated with HIV-1 (NL4.3) infected primary CD4+ T cells for 1 h at 37°C exactly as in Figure 1. Dye-labeled uninfected autologous primary target T cells were added and cells incubated for 48 h to allow for cell-cell spread of HIV-1. T cells were then fixed and stained for intracellular HIV-1 Gag and analysed by flow cytometry. **(A)** Representative dot plots showing the percentage of Gag + target cells in the absence of antibody (untreated) or in the presence of 5 μg/ml J3, J3-Fc or VRC01. 4E10 did not inhibit cell-cell transfer (50 μg/ml). NI target = uninfected target cells only. **(B)** The percentage of Gag+, dye-labeled target T cells relative to untreated controls was quantified and IC50 values (μg/ml) were calculated. A representative from experiments performed with two independent donors is shown.

Inconsistencies exist in the literature about the relative efficacy of bNabs during cell-cell spread between primary T cells. Therefore we next tested our panel of bNabs for inhibition of primary T cell-primary T cell spread using an established flow cytometry Gag transfer assay [14,41]. To do this CD4+ T cells were isolated from peripheral blood mononuclear cells, stimulated with PHA and IL-2 and infected with HIV-1 by spinoculation. After 48 h, HIV-1 infected primary CD4+ T cells were incubated with serial dilutions of antibodies and mixed with autologous, dye labeled primary CD4+ T cells and cell-cell spread quantified by flow cytometry to measure the percentage of Gag + target cells in the presence of various concentrations of bNabs. Figure 6 shows that again J3 and J3-Fc were the most inhibitory CD4 binding site antibodies tested (IC50 μg/ml = 1.5 and 1.8 respectively), with VRC01 inhibiting with an IC50 of 6.5 μg/ml, b12 of 19.3 μg/ml and somewhat surprisingly HJ16 showing reduced efficacy with an IC50 of 41.9 μg/ml. Similar to what we found with primary-Jurkat inhibition, PG9 and PG16 were effective inhibitors of cell-cell spread mediated by primary CD4 T cells (IC50 of 1.5 μg/ml and 0.4 μg/ml respectively) whereas MPER antibodies 2F5 and 4E10 were again poor inhibitors (IC50 of 41.7 μg/ml and >50 μg/ml respectively), while 2G12 also failed to block at the highest concentration tested.

### J3 and J3-Fc potently inhibit cell-cell spread from macrophages to T cells

**Figure 7:**
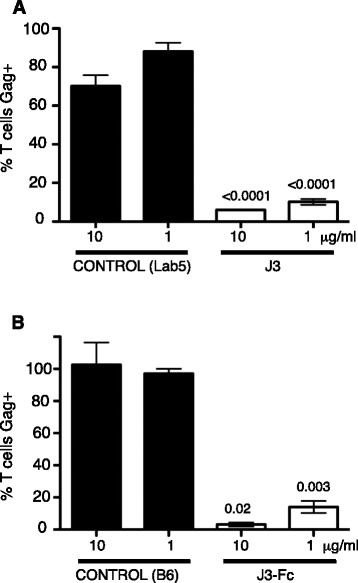
**Neutralisation of cell-cell spread from macrophages to T cells by J3 and J3-Fc. **HIV-1 (BaL) infected macrophages were incubated with the indicated concentrations of **(A)** J3, **(B)** J3-Fc and negative controls (Lab5 or B6) for 1 h at 37°C. An equal number of autologous, uninfected CD4+ T cells were then added and cells incubated for 48 h. T cells were recovered from macrophages by washing, fixed and stained for intracellular HIV-1 Gag and analysed by flow cytometry. The percentage of Gag + T cells relative to controls (Lab5 for J3 and B6 for J3-Fc) is shown. Data are the mean and SEM from 3 independent experiments. Statistical analysis was performed by Anova with Tukey’s post-test.

Cell-cell spread of HIV-1 is not restricted to dissemination of virus between T cells. Macrophages can also transmit virus by direct cell-cell spread to T cells [12,42]. Macrophages support productive infection by HIV-1 and are also considered to act as long-lived reservoirs that may contribute to viral persistence. We therefore assessed the ability of VHH J3 and J3-Fc to block cell-cell spread of HIV-1 from macrophages to primary T cells. Monocytes were purified from whole blood, differentiated into macrophages *in vitro*, infected with the macrophage-tropic HIV-1 BaL strain and co-cultured with autologous CD4+ T cells in the presence of different concentrations of J3, J3-Fc or the non-inhibitory controls Lab5 and B6. CD4+ T cells were separated from macrophages after 48 h and the proportion of Gag + T cells was quantified by flow cytometry. Figure 7 shows that the percentage of T cells which stained positive for HIV-1 Gag was significantly lower in the presence of J3 compared to the control (Lab5) at both 10 μg/ml (*p =* <0.0001) and 1 μg/ml (*p =* <0.0001). Furthermore, J3-Fc also significantly reduced spread of HIV-1 from macrophages to T cells at both 10 μg/ml (*p =* 0.02) and 1 μg/ml (*p =* 0.003). Taken together, these data show that J3 and J3-Fc potently neutralise cell-cell spread of HIV-1 between relevant human cell types including CD4+ T lymphocytes and macrophages and that that size does not appear to preclude antibody from inhibiting of macrophage-T cell spread when the antibody targets the CD4 binding site.

## Discussion

Since 2009 the discovery of the new generation of anti-HIV-1 bNabs has re-invigorated attempts to elicit protective antibodies via vaccination [3] with recent advances culminating in the development of cleaved trimeric Env that binds exclusively to bNabs [43]. In parallel, it has been shown that cell-cell HIV-1 spread is resistant to some anti-retrovirals [16,17] so it has been hypothesised that this mode of infection could be involved in the persistence of viral reservoirs in patients on therapy. Furthermore, it has been suggested that cell-cell spread of HIV-1 could be resistant to neutralising antibodies due to more temporal and/or spatial constraints imposed on the antibodies between two cells, as opposed to between a cell and budded HIV-1 virion. However, the bNab b12 has been visualised at the virological synapse [10], thus weakening the size restriction argument, although it remains plausible that the higher efficiency of cell-cell spread could render certain bNabs ineffective. Therefore efforts are underway to assess the effectiveness of bNabs during cell-cell spread and to identify antibodies capable of most effectively blocking this mode of HIV-1 spread [10,18,44,45].

In this study we have evaluated a panel of bNabs for their inhibitory activity during cell-cell spread including a number of second generation antibodies that have not been previously evaluated for inhibition of cell-cell spread of HIV-1 (J3, HJ16 and PG9). Of this group, we find that J3, a highly broadly neutralising llama antibody induced by immunisation that targets the CD4 binding site can potently neutralise cell-cell HIV-1 spread between physiologically relevant human cell types including T cells and macrophages. Furthermore this effect is not simply due to the smaller size of the VHH since J3-Fc displayed increased neutralisation of both cell-free and cell-cell spread. Different assays for cell-cell spread may necessitate the use of different cell numbers thereby making direct comparison of IC50s complicated; however, we have consistently found that while bNabs VRC01, HJ16, PG9, PG16, b12, 2G12 and 2F5 are all capable of inhibiting T cell-T cell HIV-1 spread to varying degrees, 4E10 is not. Importantly, directly comparing antibodies for inhibition of cell-cell spread between primary T cells yielded similar results and we consistently found that antibodies targeting the MPER were poor inhibitors of T cell-T cell spread mediated by primary T cells.

J3 is a highly potent inhibitor of HIV-1 and has been shown to recognise up to 96% of viral circulating strains and neutralise cell-free virus with a median IC50 of 0.9 μg/ml [22]. That J3 can also inhibit T cell-T cell and macrophage-T cell spread of HIV-1 at low concentrations (1 μg/ml) supports the development of J3 as a prophylactic agent against HIV-1. Indeed the thermal and pH stability of VHH [26] coupled with the breadth and potency of J3 makes it potentially useful as part of a microbicide gel, and a study is underway to assess the efficacy of a J3-gel in a non-human primate high dose challenge. By reconstituting the heavy-chain only J3 into an antibody format to produce J3-Fc, we demonstrated here that inhibition of cell-cell infection by J3 was not simply a product of its smaller size since J3-Fc is six times larger than J3 but was consistently better able to neutralise cell-associated and cell-free virus infection. Previous studies have shown bi-valent VHH have increased affinity for antigen and potency of neutralisation [46,47]. This increase in potency is therefore likely due to avidity effects conferred by the bi-valent nature of J3-Fc since the J3 HCAb was more potently neutralising of cell-free virus when compared to the VHH alone on a molar scale against eleven viruses from subtypes A, B, C and CRF AG and BC including transmitter/founder viruses. Thus the high affinity and resulting neutralisation potency and breadth of J3 does not result from it being smaller versions of the naturally occurring antibodies, as was the case for the Fab fragment of the human mAb 17b [48].

That llama-human hybrid VHH-Fc molecules are more potently neutralising and contain human Fc receptors enabling antibody effector functions is encouraging when considering their potential application as reagents for HIV prophylaxis mediated by passive transfer of neutralising antibodies [49]. A further useful comparison would have been with a conventional heavy and light chain J3 antibody; however, it is not possible to construct such a chimera as the VHH framework 1 signature prevents light chain interaction and altering this region would allosterically disrupt the J3-Env interface. However, our finding that the conventional patient-derived antibody HJ16 can neutralise cell-cell infection mediated by Jurkat T cells with comparable potency to J3 indicates that the additional width of a double-headed immunoglobulin is not disadvantageous in blocking cell-cell HIV-1 transmission. These data are consistent with a previous report that the Fab fragment of anti-CD4 binding site antibody b12 is less potent than the full length antibody [50].

The predominant mode of HIV-1 cell-cell spread between immune cells occurs at virological synapses. Immunofluorescence imaging has demonstrated that antibodies are readily able to access antigen at the HIV-1 virological synapse [5,6,9,10,12,13], disrupt synapse formation and reduce cell-cell spread. That the llama antibody VHH J3 and the reconstituted heavy-chain format J3-Fc can be detected at intercellular contacts formed between HIV-1 infected and uninfected T cells, and can significantly inhibit synapse formation, suggests that inhibition of cell-cell spread by J3 and J3-Fc may be mediated, at least in part, by their ability to interfere with Env-CD4 binding and impair virological synapse formation, as well as by direct neutralisation of virus during transmission across the synapse.

J3, VRC01, HJ16 and b12 all target the CD4 binding site on Env, which has been suggested to be more resistant in TZMbl cells to an antibody-mediated blockade of cell-associated virus relative to cell-free virus [18]. Comparing antibody potency between two different modes of infection is complicated when the efficiency of transmission varies as greatly as in the case of cell-cell and cell-free spread of HIV-1 [51]. Inherently, cell-cell transmission is more efficient and thus infectious units of virus are greater and pose a larger obstacle to any inhibitor. However, by directly comparing the relative potencies of different antibodies targeting the same site on HIV-1, we can also consider whether any particular site, such as the CD4 binding site, is less exposed during cell-cell spread and thus whether the groups of bNabs vary in their ability to block the same viral strain based on the mode of infection. If a specific site is less effectively targeted or less accessible to antibody during cell-cell transmission then no antibodies targeting that site should be capable of potent inhibition of this mode of infection. However, this is not what we observed for CD4 binding site antibodies during cell-cell spread. Here we found that J3 and (under most conditions) HJ16 efficiently blocked cell-cell spread of HIV-1, while VRC01 and b12 showed variable efficacy. Consistent with our results, VRC01 and b12 were reported to be relatively weak inhibitors of cell-cell spread in other studies [10,18,44,45]. The particularly weak activity of b12 may reflect variation in the binding kinetics of the different antibodies, or differences in how the boundaries of the CD4 binding site are defined; since CD4 binding site antibodies are grouped primarily for their ability to compete with soluble CD4 for binding to Env and inability to bind Env point mutants which cannot bind CD4. However, this does not mean they all bind to precisely the same footprint on Env or contact all of the same viral amino acids. For example, b12 binds some areas of Env not utilised by the other antibodies (e.g. VRC01) namely the tryptophan 100 pocket [52] and both b12 and VRC01 can bind a mutant Env that can no longer bind CD4 [23]. Thus CD4 binding site antibodies do not all interact with a single site on Env, but rather with an overlapping area with different molecular contacts contributing to each interaction [19]. This may explain why some CD4 binding site mAbs display potent inhibition of cell-cell spread whilst others do not.

We found that some antibodies specific for other epitopes in Env (glycan moieties, V1/V2 and MPER) were less efficient in blocking T cell-T cell spread and required a higher concentration to achieve 50% inhibition of cell-cell spread compared to antibodies targeting the CD4 binding site. Specifically when compared head to head across the same concentration range, antibodies targeting the MPER region (antibodies 2F5 and 4E10) do not perform as well as those targeting the CD4 binding site during cell-cell spread of this viral strain. This finding is consistent with recently published data [44]. Therefore whilst caution should be applied in extrapolating *in vitro* data to the *in vivo* situation to infer that one target may be a better or worse therapeutic strategy, our data indicate that some CD4 binding site antibodies can be highly effective inhibitors of cell to cell spread of HIV-1. Differences in the inhibitory activity of antibodies targeting related epitopes also highlights the importance of evaluating a number of inhibitors targeting the same epitope. It will also be informative in future to evaluate bNabs during cell-cell spread using a panel of primary isolates representing multiple strains and clades to consider whether viruses that may be more resistant to neutralisation are better able to exploit cell-cell spread to avoid inhibition.

The relative contribution of cell-free and cell-cell spread *in vivo* is difficult to determine. However, cell-to-cell spread mediated during physical contact between infected and uninfected cells may be an important mode of HIV-1 dissemination in lymphoid tissues, where CD4+ T lymphocytes are densely-packed and likely to frequently interact. In support of this, recent intravital imaging studies have validated the concept of the virological synapse *in vivo* [53,54]. Because cell-cell spread has been shown to be orders of magnitude more efficient than equivalent cell-free infection [9-14], it is reasonable to hypothesise that less efficient antibody-mediated neutralization of cell-cell spread by some NAbs may impact on viral replication *in vivo* [53,54].

It is worth noting that evaluation of bNabs is commonly performed using the standardised HeLa TZM-bl assay in which neutralising activity of antibodies is tested against different HIV Envs by generating pseudotyped viruses in 293T cells that are then used in cell-free infection assays. Our data reveal interesting differences in the ranking of antibodies during cell-free infection using the TZM-bl assay when compared to cell-cell spread. For example J3 was the most potent of the anti-CD4 binding site antibodies against HIV-1 cell-free infection and maintained its ranking as a potent inhibitor when evaluated against other antibodies during cell-cell infection. By contrast, b12 might be predicted to perform well in cell-cell spread based on the similarity in IC50s values between b12, VRC01 and HJ16 in cell-free titrations, but this was not what we found. Other antibodies such as 4E10 performed well in cell-free evaluation against the HIV-1 strain NL4.3 using the standardised TZM-bl assay but were essentially inactive during cell-cell spread. Thus the relative potency with which a given antibody will inhibit cell-cell spread may not necessarily be predicted by its activity against cell-free virus when evaluated via the standardised TZM-bl assay. We propose that valuable insight could be gained by comparing antibodies targeting the same site for the ability to inhibit cell-cell infection using physiologically relevant primary human cells in order to identify potent inhibitors of cell-cell spread. Since it is well established that cell-cell infection is a significantly more efficient mode of virus spread this may pose a more stringent challenge to inhibition, but such endeavours could help to identify the most effective prophylactic antibodies for *in vivo* use to target HIV-1 during the course of natural infection.

## Conclusions

In conclusion, we have tested a panel of bNabs targeting different sites on Env for their ability to block cell-cell HIV-1 infection including a number not previously evaluated for inhibition of cell-cell spread. We identify the VHH J3 as a particularly efficient inhibitor of both cell-free and cell-cell spread and show that the potency of J3 is not simply a function of its smaller size, since J3-Fc was consistently more inhibitory. The ability of J3 and J3-Fc to inhibit cell-cell spread of HIV-1 between T cells and from macrophages to T cells at concentrations within the low μg/ml range, coupled with the thermal and pH stability of VHH, provides strong support for the development of J3 as a novel therapeutic with studies underway to assess the efficacy of a J3-gel in a non-human primate high dose challenge.

## Methods

### Viruses

The following HIV-1 molecular clones or viruses were originally obtained from the NIH AIDS Reagent and Reference Program (ARRP, NIH, USA): HIV-1 molecular clone pNL4.3 produced by M. Martin; BaL virus donated by S. Gartner, M. Popovic and R. Gallo; transmitter/founder virus clones CH058 and CH077 obtained from Dr. John Kappes and Dr. Christina Ochsenbauer [55,56]; and the subtype B and C HIV-1 Reference Panels of Env Clones [57,58]. The 96ZM651.02 gp160 clone was kindly provided by Dr D. Montefiori (Duke University Medical Center, Durham, NC) through the Comprehensive Antibody Vaccine Immune Monitoring Consortium (CA2 VIMC) as part of the Collaboration for AIDS Vaccine Discovery (CAVD). Virus was prepared from molecular clones by transfection of 293T cells and virus titrated on HeLa-TZM-bl reporter cells using the Bright-Glo Luciferase assay kit (Promega) to calculate the viral titer (TCID50/ml). HIV-1 envelope pseudotyped viruses were produced in 293T cells by co-transfection with the pSG3Δenv plasmid and virus titrated on HeLa-TZM-bl reporter cells as above. BaL virus was prepared by passage in human peripheral blood mononuclear cells and was a gift from P. Mlcochova. All cell-cell transmission experiments were performed using the HIV-1 strain NL4.3 except for macrophage - T cell transmission assays that were performed using HIV-1 BaL.

### Cells

HeLa-TZM-bl cells were obtained from the Centre for AIDS Reagents, National Institutes of Biological Standard and Control, UK (CFAR, NIBSC) and donated by J. Kappes, X. Wu and Tranzyme Inc. and cultured in Dulbecco’s Modified Eagle’s Medium (DMEM) supplemented with streptomycin (100 μg/ml), penicillin (100 U/ml) and 10% fetal calf serum (FCS, Invitrogen). The CD4+/CXCR4+ T cell line Jurkat CE6.1 and derivative Jurkat line 1G5 (obtained through the ARRP NIH: from Dr. Estuardo Aguilar-Cordova and Dr. John Belmont) were maintained in RPMI 1640 supplemented with streptomycin (100 μg/ml), penicillin (100 U/ml) and 10% fetal calf serum (Invitrogen). Primary CD4+ T cells were isolated from peripheral blood mononuclear cells by Ficoll-Hypaque gradient, stimulated with 1 μg/ml PHA (Sigma) and 10 IU/ml IL-2 (CFAR, NIBSC, UK) and CD4^+^ T cells isolated by negative selection according to manufacturer’s instructions (Miltenyi Biotec). This routinely gave >90% pure CD4+ T cells. Primary CD4+ T cells were maintained in RPMI 1640 supplemented with streptomycin (100 μg/ml), penicillin (100 U/ml), 20% fetal calf serum (FCS, Invitrogen) and 10I U/ml IL-2. Macrophages were purified from peripheral blood mononuclear cells by Ficoll-Hypaque gradient and monocytes were allowed to adhere to tissue culture treaded plates. Non-adherent cells were removed and autologous CD4+ T cells were purified as described above. Monocytes were seeded in 48 well trays, differentiated to macrophages with M-CSF and were maintained in RPMI supplemented with 10% human serum (Sigma).

### Antibodies

PG9, PG16, b12 and b6 were kind gifts from Dennis Burton, VRC01 was a kind gift from John Mascola and HJ16 was a kind gift from Davide Corti and Antonio Lanzavecchia. 2G12, 2F5, 4E10 were obtained from CFAR NIBSC and were originally donated by H Katinger (POLYMUN Scientific GMBH). J3 has been described previously [22].

### VHH purification and construction of full-length J3-Fc HCAB antibody

Expression from the pCAD51 vector incorporates a 6-His and a c-Myc tag to the C terminus of the VHH and removes the bacteriophage gene III product. The VHH were purified by means of the attached His tag using TALON Metal Affinity Resin (Takara Bio Inc.) as described previously [22]. DNA encoding J3-Fc was generated using overlapping PCR with J3 and human immunoglobulin G1 Fc (including the hinge and CH2 and CH3 domains) as templates. The resulting fragment was ligated into an expression vector and produced by transient transfection in HEK 293 cells and purified using Protein A affinity chromatography.

### TZM-bl neutralisation assay

The neutralising activity of antibodies was assayed in duplicate in the TZM-bl cell-based assay against NL4.3 produced by 293T cells [57,59]. Infection of cells was detected using with Bright-Glo luciferase reagent (Promega) using a Glomax plate reader (Promega). IC50 titers were calculated using the XLFit4 software (IDBS).

### T cell-T cell inhibition assay

Jurkat and primary CD4+ T were infected by spinoculating at 1200 × g for 2 h at an MOI of 0.1-0.2 (calculated using the TCID50 obtained by titrating virus on TZM-bl cells). Cells were phenotyped for surface Env expression using 2G12 (POLYMUN) followed by anti-human immunoglobulin G-phycoerythrin (Jackson Immunoresearch). Intracellular Gag staining was performed by fixing cells in 4% formaldehyde, permeabilising in BD Perm/Wash buffer (Becton Dickinson) and HIV-1 Gag was detected using the PE-conjugated antibody RC57-RD1 (Coulter). The percentage of Gag + and Env + cells was quantified by flow cytometry. To quantify cell-cell spread, 1×10^4^ HIV-1 infected T cells were preincubated with serial dilutions of bNAbs and controls for 1 h at 37°C and mixed with 2.5×10^5^ 1G5 Jurkat T cells for 24 h at 37°C. Cells were then washed and luciferase activity in the 1G5 target cells was measured by luminescence assay using the Bright-Glo luciferase assay (Promega).

Alternatively, 2×10^5^ HIV-1 infected Jurkat cells were incubated with bNabs for 1 h 37°C and mixed with 1G5 Jurkat T cells in a ratio of 1:4 and at various times post-mixing the cells were harvested and genomic DNA extracted (Qiagen) and quantitative real-time PCR was performed to measure cell-cell spread of HIV-1 as described previously [9]. To determine the relative contribution of cell-cell and cell-free spread to the luciferase signal in 1G5 target cells over 24 h, HIV-1 infected and uninfected cells were incubated as described above (without bNabs) and the luciferase signal from cell-cell spread was measured by luminescence. To quantify cell-free infection in parallel, the same numbers of HIV-1 infected cells were incubated alone without the addition of target cells for 24 h in order to allow cell-free virus release. Virus-containing supernatants were harvested and incubated with 1G5 target cells for another 24 h to allow cell-free infection and luciferase activity was measured by luminescence.

To measure inhibition of cell-cell spread between autologous primary CD4+ T cells by bNabs a flow cytometry Gag transfer assay was used [14,41]. Briefly, CD4+ T were purified from activated PBMCs as described above and infected by spinoculation. Forty-eight hours later 5×10^5^ HIV-1 infected primary CD4+ T cells were incubated with bNabs for 1 h 37°C and mixed with 1×10^6^ of autologous target CD4+ T cells that were prelabeled with CellTrace Far Red dye. Forty-eight hours later cells were stained for intracellular HIV-1 Gag as described above and the percentage of Gag+ dye-labeled target cells was determined by flow cytometry analysis.

### Macrophage-T cell inhibition assay

Quantification of cell-cell spread from macrophages to T cells was performed essentially as described previously with minor modifications [12,51]. Briefly, 2 × 10^5^ macrophages per well were infected with HIV-1 BaL (30 ng of p24 per well) for 6 h at 37°C, washed and incubated for 7 days. Macrophages were then incubated with J3, J3-Fc or a relevant non-specific control (VHH Lab5 for J3 and B6 for J3-Fc) for 1 h at 37°C after which time 4 × 10^5^ autologous CD4+ T cells (purified and activated with PHA and IL-2 as described above) were added to each well. CD4+ T cells were harvested 48 h later by washing, stained for intracellular HIV-1 Gag as described above and analysed by flow cytometry.

### Immunofluorescence microscopy

HIV-1 infected T cells were washed in RPMI1%-FCS, mixed with an equal number of uninfected target T cells and incubated on poly-L-lysine (Sigma) treated coverslips at 37°C for 1 h in the presence of the VHH J3, J3-Fc or control antibodies (Lab5 or B6) according to published methods [5]. The cells were fixed in 4% formaldehyde in PBS-1% BSA for 15 min, washed and permeabilised in 0.1% Triton X-100/5% FCS and HIV-1 Gag was stained with rabbit antisera against Gag p17 and p24 (donated by G. Reid and obtained from the CFAR, NIBSC, UK). Primary antibodies were detected with anti-rabbit Cy3 (Gag) (Jackson Immunoresearch), anti-human FITC (J3-Fc, B6) (Jackson Immunoresearch), or mouse anti-Myc antibody (J3, Lab5) (Millipore) followed by anti-mouse Alexa-488 (Invitrogen). All secondary antibodies were tested for an absence of inter-species reactivity. Coverslips were mounted with ProLong antifade mounting solution (Invitrogen) and imaged using a DeltaVision ELITE Image Restoration Microscope (Applied Precision/Olympus) through a 60× 1.4 NA oil immersion lens with an inverted Olympus IX71 microscope and a CoolSNAP HQ2 camera. Images were deconvolved with softWoRx 5.0 and processing was performed using Huygens Professional version 4.0 and Adobe Photoshop CS3. Random fields were selected and the number of contacts between HIV-1 Gag + T cells and uninfected T cells that had formed virological synapses (defined by polarisation of HIV-1 Gag to the cell-cell interface) was quantified.
